# Differential Prognostic Indicators for Locoregional Recurrence, Distant Recurrence, and Death of Breast Cancer

**DOI:** 10.1155/2013/946945

**Published:** 2013-11-26

**Authors:** Rungnapa Chairat, Adisorn Puttisri, Asani Pamarapa, Jirause Moollaor, Chamaiporn Tawichasri, Jayanton Patumanond

**Affiliations:** ^1^Clinical Epidemiology Unit, Faculty of Medicine, Chiang Mai University, Chiang Mai 50200, Thailand; ^2^Department of Nursing, Uttaradit Hospital, Uttaradit 53000, Thailand; ^3^Department of General Surgery, Nakhon Sawan General Hospital, Nakhon Sawan 60000, Thailand; ^4^Department of General Surgery, Uttaradit General Hospital, Uttaradit 53000, Thailand; ^5^Department of General Surgery, Lampang General Hospital, Lampang 52000, Thailand; ^6^Clinical Epidemiology Society, Chiang Mai 50200, Thailand

## Abstract

*Objective*. To explore prognostic characteristics for locoregional recurrence, distant recurrence, and mortality in patients with breast cancer. *Methods*. A 5-year retrospective review of patients was conducted in two university affiliated hospitals in the north of Thailand. Prognostic characteristics and clinical outcomes were retrieved from medical registry. Death was verified by the civil database from the Ministry of Interior, direct telephone contact, or by prepaid postcard. Data were analyzed by stratified Cox's regression proposed by Lunn & McNeil, in which multiple-typed outcomes were analyzed in a single multivariable model. *Results*. The assembled cohort comprised 829 patients. Under the multivariable analysis, 7 prognostic characteristics were significant prognostic indicators. Positive axillary lymph nodes >3 and presence of lymphovascular invasion (LVI) increased locoregional recurrence, while disease stage 3, positive axillary lymph nodes >3, and radiotherapy increase distant recurrence. Hormonal therapy reduced the distant recurrence. Pathological tumor size >2 cm, disease stage 3, positive axillary lymph nodes >3, and presence of LVI increased, while hormonal therapy and chemotherapy reduced death. *Conclusions*. Clinical characteristic reflecting tumor invasions increased locoregional recurrence, distant recurrence, or death, while hormonal therapy and chemotherapy reduced such risks. The effect of radiation remained inconclusive but may increase the risk of distant recurrence.

## 1. Introduction

Breast cancer is a common medical problem and one of the leading causes of death among malignant conditions in women worldwide [1]. The incidence of breast cancer varied geographically. In Thailand it is the leading cancer among females with continuously increasing trend. The incidence increased from 37.9 in 2007 to 53.8 per 100000 population in 2011. The mortality also rose from 6.8 to 8.4 per 100000 population in the corresponding years [2].

Despite medical progresses in diagnosis and treatments in the past 10 years, recurrence after complete treatment was still common, both locoregional (27%) and distant (11%) [3, 4]. Survival and mortality of breast cancer varied from study to study [3, 5], and the most common cause of death was distant metastasis [5].

One of the most challenging tasks in managing breast cancer cases was disease prognostication of patients who were likely to have recurrence as prompt detection of recurrence may save the patients' life or increase the survival time [6]. This should be done by risk evaluation of recurrence based on knowledge, genetics, molecular, biochemical sciences, and cytology [7]. With medical resource limitation, clinicians in poor developing countries were forced to manage patients with additional treatments, in which many cases might have been unnecessary, resulting in overtreatment [8].

Previous study mentioned demographic, pathological characteristics, hormone receptors, and types of treatment as the main prognostic factors for recurrence and survival of patients with breast cancer and most studies were done in developed countries [9, 10]. Whether these characteristics would be similar in poor developing countries is still inconclusive [11]. Very few studies have demonstrated whether the prognostic characters for different types of disease progression are different.

We conduct the present study to identify patients characteristics that may be used by clinicians as prognostic determinants for breast cancer progression, locoregional recurrence, distant recurrence, and death. Clinicians may use these determinants to help disease prognostication or to help guiding patient managements or monitoring.

## 2. Subject and Methods

### 2.1. Subject and Setting

The 5-year medical files of women with documentary confirmed breast cancer diagnosis from two university affiliated hospitals in Lampang and Uttaradit in the north of Thailand during 2006 and 2010 were reviewed. We assembled a cohort of patients who were still free of locoregional or distant recurrence. These patients were documentary traced for local, distant recurrence and death from breast cancer.

### 2.2. Data Sources and Collection

#### 2.2.1. Key Information

The focusing study characteristics included patient characteristics, age, types of surgery; pathological characteristics, tumor size, histological type, histological grade, stage, dissected axillary lymph nodes, positive axillary lymph nodes, and lymphovascular invasion (LVI); receptor status, ER receptor, PR receptor, and HER2 receptor; and types of treatments, radiotherapy, hormonal therapy, and chemotherapy. Main clinical outcomes of interest, locoregional recurrence, distant recurrence, and death, were verified from medical registry. Survival status of patients who lost to followup was also verified by civil database retrievable from the Ministry of Internal Affairs and through prepaid postcard or direct contact through telephone.

### 2.3. Data Analysis

The patient characteristics were compared among the three different types of disease progression (locoregional recurrence, distant recurrence, and death from breast cancer). Prognostic indicators for each of the three clinical outcomes were analyzed as univariable and multivariable hazard ratio by stratified Cox's regression based on Lunn & McNeil's methods, in which multiple prognostic characteristics could be computed for multiple types of outcomes in only one single regression model [12]. The direction of significant prognostic characteristics were presented and summarized.

### 2.4. Ethical Approval

This retrospective data analysis was approved by the research ethical committees of Lampang Hospital and Uttaradit Hospital and the research ethical committee of the Faculty of Medicine, Chiang Mai University.

## 3. Results

A cohort of 829 patients with postoperative breast cancer was assembled. Most characteristics of patients with the three different clinical outcomes were significantly different, except for types of surgery, histological type, dissected axillary lymph nodes, and HER2 receptor (Table 1).

Under the univariable analysis, most of the characteristics were significant (Table 2). With multivariable analysis using stratified Cox's regression based on Lunn & McNeil's methods, there were only 7 significant characters. These characteristics were pathological tumor size >2 cm, stage 3, positive axillary lymph nodes >3, lymphovascular invasion (LVI), radiotherapy, hormonal therapy, and chemotherapy (Table 3). Summarized direction of significant prognostic hazards in breast cancer (Table 4).

Positive axillary lymph nodes >3 (HR = 2.43, 95%  CI = 1.35–4.37,  *P* = 0.003) and presence of LVI (HR = 2.01, 95%  CI = 1.09–3.71,  *P* = 0.026) increased the hazard of locoregional recurrence.

Stage 3 (HR = 2.44, 95%  CI = 1.57–3.80,  *P* < 0.001), positive axillary lymph nodes >3 (HR = 1.79, 95%  CI = 1.10–2.93,  *P* = 0.020), and radiotherapy (HR = 2.40, 95%  CI = 1.50–3.84,  *P* < 0.001) increased the hazard for distant recurrence, while hormonal therapy (HR = 0.44, 95%  CI = 0.30–0.65,  *P* < 0.001) reduced the hazard.

For death from cancer, pathological tumor size >2 cm (HR = 1.56, 95%  CI = 1.02–2.40,  *P* = 0.040), stage 3 (HR = 2.17, 95%  CI = 1.42–3.32,  *P* < 0.001), positive axillary lymph nodes >3 (HR = 2.16, 95%  CI = 1.36–3.43,  *P* = 0.001), and presence of LVI (HR = 2.87, 95%  CI = 1.69–4.87,  *P* < 0.001) increased the hazard, while hormonal therapy (HR = 0.57, 95%  CI = 0.40–0.83,  *P* = 0.003) and chemotherapy (HR = 0.27, 95%  CI = 0.14–0.52,  *P* < 0.001) reduced the hazard (Table 3).

## 4. Discussion

It was well realized that breast cancer is a heterogeneous disease originating from multiple somatic mutations, resulting in different risk for recurrence after treatment, which was commonly observed in patients with the same clinical stages [13].

### 4.1. Prognostic Characteristics for Locoregional Recurrence

#### 4.1.1. Positive Axillary Lymph Nodes >3 and Lymphovascular Invasion

There were 2 proposed hypotheses mentioning locoregional recurrence of breast cancer. Locoregional recurrence of breast cancer could be a sign of disseminated disease from the beginning [14, 15] and cancer cell might have already invaded into the blood stream when breast masses were detected or might have invaded through lymph vessels into the lymph nodes. On the other hand it might be associated with incomplete removal of cancer cell, resulting in residual tumor [16]. The present findings agreed with previous studies reporting positive axillary lymph nodes >3 and lymphovascular invasion as prognostic factors for locoregional recurrence [9, 17].

### 4.2. Prognostic Characteristics for Distant Recurrence

#### 4.2.1. Disease Stage 3

Stage 3 breast cancer involved axillary lymph nodes. The chances of disseminating to other distant organs were increased [18]. This was also mentioned by other studies [19].

#### 4.2.2. Positive Axillary Lymph Nodes >3

Distant dissemination of cancer cells occurred from spreading of disease from axillary lymph nodes involvement [20].

#### 4.2.3. Radiation Therapy

After surgical removal of cancer, some patients would be irradiated as indicated. The association with an increase in risk of distant recurrence was confounded by indication or contraindication as mentioned above. It was likely that patients who were selected for irradiation were those with poor prognosis (confounded by indication). On the contrary, irradiation might stimulate production of miR-21 in the cell cycle resulting in radiation resistance and promoting cancer cell proliferation, which caused invasion and dissemination to other organs [21]. There were studies explaining an association between irradiation and an increase in risk of distant recurrence by alteration of cancer cells phenotype and an increase in breast cancer stem cells (BCSC) causing cancer cells more aggressive [22–24]. Recently, one study proposed an increase in awareness of irradiation in patients with early stage breast cancer [25]. In the present study, after exclusion of patients with stage 3 (to whom radiation was routinely recommended), analysis in only patients with stages 1 and 2 demonstrated that radiation therapy increased the hazard of distant recurrence (HR = 3.83, 95%  CI = 2.15–6.80,  *P* < 0.001). Although the present study agreed with the aforementioned study, many previous studies reported the opposite findings that irradiation reduced the risk of recurrence [3] and spreading to other organs [26].

#### 4.2.4. Hormonal Therapy

The most common hormone therapy, tamoxifen, is a competitive partial agonist-inhibitor for estrogen receptor located on cancer cells. Its effect on cell proliferation inhibition results in a reduced risk of dissemination to other organs [27].

### 4.3. Prognostic Characteristics for Death from Cancer

Patients who died of breast cancer were caused by consequences of distant recurrence or metastasis [19]. It could also be restated that distant recurrences are the surrogates of death [26].

#### 4.3.1. Pathological Tumor Size >2 cm

Tumors larger than 2 cm were more likely to spread to adjacent lymph nodes, increasing the chance of spreading to other organs [28], which led to an increasing risk of death.

#### 4.3.2. Stage 3 and Positive Axillary Lymph Nodes >3

This could be due to the common findings that stage 3 and positive axillary lymph nodes >3 increase the chance of spreading to distant organs, which is the cause of death.

#### 4.3.3. Presence of LVI

Direct spreading to lymph vessels increased the chance of spreading to other organs [29]. There were studies that confirmed such association [30].

#### 4.3.4. Hormonal Therapy

As mentioned above, treatment with tamoxifen reduced the chance of spreading to distant organs [27], resulting in a reduced hazard of death.

#### 4.3.5. Chemotherapy Therapy

Most chemotherapy was aimed at micrometastatic cell. This action reduced tumor cell dissemination to distant organs which was the cause of death [31, 32].

In summary, the prognostic characteristics for breast cancer recurrence and death reported in the present study were mostly similar to previous reports [9, 10]. However, it should be noticed that the effect of radiation therapy was unexpectedly opposite. It could be argued that this was likely due to the “confounding by indication” effect. Nevertheless recent animal studies and reports in human are discussing the probability that irradiation of tumor cells stimulates breast cancer stem cell (BCSC) to proliferate and form radioresistance [22]. At present, there are no researches conducted in human to clarify this hypothesis. The present study may be one of the earliest observational reports on this finding. Whether radiation therapy is beneficial to breast cancer patients remains the subject of further researches in the future.

## 5. Conclusions

Evidences of tumor cells invasions, positive axillary lymph nodes >3 and presence of LVI, increased the risk of locoregional recurrence. The same evidence, disease stage 3 and positive axillary lymph nodes >3, increased the risk of distant recurrence, while hormonal therapy reduced the risk. The risk of death from cancer was increased in pathological tumor size >2 cm, disease stage 3, positive axillary lymph nodes >3, and presence of LVI, while hormonal therapy and chemotherapy therapy reduced such risk. The effect of radiation is inconclusive but may increase the risk of distant recurrence.

## Figures and Tables

**Table 1:** Clinical profiles and treatment of breast cancer (*n* = 829), by first occurring events.

Characteristics			Recurrence						*P* value
	Event-free (*n* = 637)		Locoregional (*n* = 83)		Distant (*n* = 78)		Death (*n* = 31)		
	*n*	%	*n*	%	*n*	%	*n*	%	
Age (year)									
≤45	166	26.1	25	30.1	30	38.5	4	12.9	0.030
>45	471	73.9	58	69.9	48	61.5	27	87.1	
Types of surgery									
MRM	543	85.2	72	86.8	70	89.7	26	83.9	0.120
Simple mastectomy	40	6.3	9	10.8	5	6.4	4	12.9	
BCS	54	8.5	2	2.4	3	3.9	1	3.2	
Pathological tumor size (cm)									
≤2	272	42.7	23	27.7	16	20.5	10	32.3	<0.001
>2	365	57.3	60	72.3	62	79.5	21	67.7	
Histological type									
Ductal carcinoma	590	92.6	82	98.8	77	98.7	30	96.8	0.191
Lobular carcinoma	5	0.8	0	0.00	0	0.00	0	0.00	
Other types	42	6.6	1	1.2	1	1.3	1	3.2	
Histological grade									
I	110	19.2	8	9.9	9	12.0	2	7.4	0.027
II	280	48.9	46	56.8	38	50.7	9	33.3	
III	183	31.9	27	33.3	28	37.3	16	59.3	
Stage									
1	218	34.2	16	19.3	9	11.5	3	9.7	<0.001
2	353	55.4	43	51.8	41	52.6	22	71.0	
3	66	10.4	24	28.9	28	35.9	6	19.3	
Dissected axillary lymph nodes									
<10	125	20.3	20	25.6	17	23.0	7	24.1	0.641
≥10	490	79.7	58	74.4	57	77.0	22	75.9	
Positive axillary lymph nodes									
≤3	530	86.0	42	53.9	38	51.4	20	69.0	<0.001
>3	86	14.0	36	46.1	36	48.6	9	31.0	
LVI									
Absent	377	60.1	23	29.5	22	29.0	11	36.7	<0.001
Present	250	39.9	55	70.5	54	71.0	19	63.3	
Receptor status									
ER receptor									
Positive	398	63.6	44	55.0	36	47.4	20	66.7	0.027
Negative	228	36.4	36	45.0	40	52.6	10	33.3	
PR receptor									
Positive	351	56.0	42	52.5	30	39.5	18	60.0	0.047
Negative	276	44.0	38	47.5	46	60.5	12	40.0	
HER2 receptor									
Positive	236	40.5	38	54.3	35	49.3	11	39.3	0.093
Negative	347	59.5	32	45.7	36	50.7	17	60.7	
Treatment									
Radiotherapy									
Yes	199	31.3	41	49.4	52	66.7	8	26.7	<0.001
No	436	68.7	42	50.6	26	33.3	22	73.3	
Hormonal therapy									
Yes	418	65.6	48	57.8	38	48.7	23	74.2	0.011
No	219	34.4	35	42.2	40	51.3	8	25.8	
Chemotherapy									
Yes	547	86.3	80	96.4	73	93.6	19	63.3	<0.001
No	87	13.7	3	3.6	5	6.4	11	36.7	

MRM: modified radical mastectomy; BCS: breast-conserving surgery; LVI: lymphovascular invasion; ER: estrogen receptor; PR: progesterone receptor; HER2: human epidermal growth factor receptor 2.

**Table 2:** Effect (hazard ratio, HR) of clinical profiles and treatment on locoregional recurrence, distance recurrence, and death in breast cancer (*n* = 829), univariable analysis.

Prognostic factors	Locoregional recurrence		Distant recurrence		Death	
	HR (95%CI)	*P* value	HR (95%CI)	*P* value	HR (95%CI)	*P* value
Age (year)						
>45 versus ≤45	0.91 (0.57–1.46)	0.696	0.64 (0.44–0.94)	0.022	1.01 (0.69–1.49)	0.942
Types of surgery						
Simple mastectomy and BCS versus MRM	0.85 (0.62–2.21)	0.609	1.41 (0.73– 2.70)	0.305	1.08 (0.62–1.88)	0.792
Pathological tumor size (cm)						
>2 versus ≤2	1.85 (1.14–2.99)	0.012	1.99 (1.30–3.04)	0.001	2.12 (1.42–3.16)	<0.001
Histological type						
Ductal CA versus lobular CA and other types	5.04 (0.70– 36.19)	0.108	3.32 (0.82–13.44)	0.093	2.40 (0.76–7.54)	0.135
Histological grade						
II and III versus I	2.21 (1.06–4.59)	0.033	1.84 (1.01–3.35)	0.047	2.04 (1.15–3.63)	0.015
Stage						
3 versus 1 and 2	3.56 (2.21–5.75)	<0.001	5.06 (3.43–7.46)	<0.001	4.53 (3.14–6.52)	<0.001
Dissected axillary lymph nodes						
≥10 versus <10	0.81 (0.49–1.34)	0.408	0.86 (0.55–1.35)	0.514	0.81 (0.54–1.21)	0.297
Positive axillary lymph nodes						
>3 versus ≤3	4.53 (2.89–7.10)	<0.001	4.93 (3.35–7.27)	<0.001	4.85 (3.39–6.92)	<0.001
LVI						
Present versus absent	3.42 (2.11–5.58)	<0.001	3.66 (2.40–5.59)	<0.001	4.35 (2.88–6.57)	<0.001
Receptor status						
ER receptor						
Negative versus positive	1.49 (0.96–2.31)	0.079	2.05 (1.40–2.99)	<0.001	1.64 (1.15–2.34)	0.006
PR receptor						
Negative versus positive	1.24 (0.80–1.92)	0.339	1.84 (1.25–2.71)	0.002	1.57 (1.10–2.24)	0.012
HER2 receptor						
Negative versus positive	0.60 (0.37–0.96)	0.033	0.89 (0.60–1.32)	0.553	0.80 (0.55–1.15)	0.225
Treatment						
Radiotherapy						
Yes versus no	2.17 (1.42–3.35)	<0.001	4.35 (2.93–6.47)	<0.001	2.72 (1.91–3.86)	<0.001
Hormonal therapy						
Yes versus no	0.64 (0.41–0.99)	0.044	0.45 (0.31–0.65)	<0.001	0.58 (0.41–0.82)	0.002
Chemotherapy						
Yes versus no	3.78 (1.19–11.96)	0.024	2.99 (1.22–7.34)	0.017	0.97 (0.57–1.64)	0.907

MRM: modified radical mastectomy; BCS: breast-conserving surgery; LVI: lymphovascular invasion; ER: estrogen receptor; PR: progesterone receptor; HER2: Human Epidermal Growth Factor Receptor 2.

**Table 3:** Effect (hazard ratio, HR) of clinical profiles and treatment on locoregional recurrence, distance recurrence, and death in breast cancer (*n* = 829), multivariable analysis (reduced model).

Prognostic factors	Locoregional recurrence		Distant recurrence		Death	
	HR (95% CI)	*P* value	HR (95% CI)	*P* value	HR (95% CI)	*P* value
Age (year)						
>45 versus ≤45	1.07 (0.64–1.78)	0.801	0.75 (0.50–1.13)	0.164	0.93 (0.62–1.40)	0.722
Pathological tumor size (cm)						
>2 versus ≤2	1.46 (0.87–2.45)	0.148	1.26 (0.80–1.97)	0.316	1.56 (1.02–2.40)	0.040
Stage						
3 versus 1 and 2	1.65 (0.94–2.91)	0.081	2.44 (1.57–3.80)	<0.001	2.17 (1.42–3.32)	<0.001
Positive axillary lymph nodes						
>3 versus ≤3	2.43 (1.35–4.37)	0.003	1.79 (1.10–2.93)	0.020	2.16 (1.36–3.43)	0.001
LVI						
Present versus absent	2.01 (1.09–3.71)	0.026	1.64 (0.96–2.83)	0.073	2.87 (1.69–4.87)	<0.001
Treatment						
Radiotherapy						
Yes versus no	0.90 (0.54–1.53)	0.709	2.40 (1.50–3.84)	<0.001	1.32 (0.86– 2.03)	0.201
Hormonal therapy						
Yes versus no	0.70 (0.44–1.11)	0.128	0.44 (0.30–0.65)	<0.001	0.57 (0.40–0.83)	0.003
Chemotherapy						
Yes versus no	1.51 (0.45–5.02)	0.502	1.36 (0.41–4.49)	0.612	0.27 (0.14–0.52)	<0.001

LVI: lymphovascular invasion.

**Table 4:** Summarized direction of significant prognostic hazards (locoregional recurrence, distance recurrence, and death in breast cancer).

Prognostic factors	Prognostic hazards		
	Locoregional recurrence	Distant recurrence	Death
Pathological tumor size >2 cm			Increase
Stage 3		Increase	Increase
Positive axillary lymph nodes >3	Increase	Increase	Increase
LVI present	Increase		Increase
Treatments			
Radiotherapy		Increase	
Hormonal therapy		Decrease	Decrease
Chemotherapy			Decrease

LVI: lymphovascular invasion.
